# ChatGPT and Google Assistant as a Source of Patient Education for Patients With Amblyopia: Content Analysis

**DOI:** 10.2196/52401

**Published:** 2024-08-15

**Authors:** Gloria Wu, David A Lee, Weichen Zhao, Adrial Wong, Rohan Jhangiani, Sri Kurniawan

**Affiliations:** 1 University of California, San Francisco School of Medicine San Francisco, CA United States; 2 McGovern Medical School University of Texas Health Science Center at Houston Houston, CA United States; 3 College of Biological Sciences University of California, Davis Davis, CA United States; 4 Department of Computational Media University of California, Santa Cruz Santa Cruz, CA United States

**Keywords:** ChatGPT, Google Assistant, amblyopia, health literacy, American Association for Pediatric Ophthalmology and Strabismus, pediatric, ophthalmology, patient education, education, ophthalmologist, Google, monitoring

## Abstract

**Background:**

We queried ChatGPT (OpenAI) and Google Assistant about amblyopia and compared their answers with the keywords found on the American Association for Pediatric Ophthalmology and Strabismus (AAPOS) website, specifically the section on amblyopia. Out of the 26 keywords chosen from the website, ChatGPT included 11 (42%) in its responses, while Google included 8 (31%).

**Objective:**

Our study investigated the adherence of ChatGPT-3.5 and Google Assistant to the guidelines of the AAPOS for patient education on amblyopia.

**Methods:**

ChatGPT-3.5 was used. The four questions taken from the AAPOS website, specifically its glossary section for amblyopia, are as follows: (1) What is amblyopia? (2) What causes amblyopia? (3) How is amblyopia treated? (4) What happens if amblyopia is untreated? Approved and selected by ophthalmologists (GW and DL), the keywords from AAPOS were words or phrases that deemed significant for the education of patients with amblyopia. The “Flesch-Kincaid Grade Level” formula, approved by the US Department of Education, was used to evaluate the reading comprehension level for the responses from ChatGPT, Google Assistant, and AAPOS.

**Results:**

In their responses, ChatGPT did not mention the term “ophthalmologist,” whereas Google Assistant and AAPOS both mentioned the term once and twice, respectively. ChatGPT did, however, use the term “eye doctors” once. According to the Flesch-Kincaid test, the average reading level of AAPOS was 11.4 (SD 2.1; the lowest level) while that of Google was 13.1 (SD 4.8; the highest required reading level), also showing the greatest variation in grade level in its responses. ChatGPT’s answers, on average, scored 12.4 (SD 1.1) grade level. They were all similar in terms of difficulty level in reading. For the keywords, out of the 4 responses, ChatGPT used 42% (11/26) of the keywords, whereas Google Assistant used 31% (8/26).

**Conclusions:**

ChatGPT trains on texts and phrases and generates new sentences, while Google Assistant automatically copies website links. As ophthalmologists, we should consider including “see an ophthalmologist” on our websites and journals. While ChatGPT is here to stay, we, as physicians, need to monitor its answers.

## Introduction

Amblyopia is a global public health problem with multiple impacts on vision and quality of life [1]. Amblyopia, if untreated, leads to permanent visual impairment in adults. The underlying causes are undiscovered anisometropia (unequal refractive error between the 2 eyes), visual deprivation such as congenital or juvenile cataracts, or strabismus with anisometropia. Treatment can be as simple as a pair of glasses. Treatment may mean occlusion therapy of the stronger eye, ensuring that the “weaker” or amblyopic eye gets visual stimulation. If treated early, the young child will have improved binocular vision.

Amblyopia is a common visual complaint in children in an ophthalmology setting. Vision at the level of 20/80 to 20/200 makes up almost 40% of the cases in a population-based study in Australia [2]. The critical period of human visual development is in the first decade of life [3]. Recent neuroplasticity models in humans and primates suggest that there is residual plasticity that extends into later years of adult life [3].

The quality of life for people with amblyopia can involve difficulties in daily activities due to impaired reading speed, stereo acuity, motor skills, motion perception, and fixation stability [4,5].

Health education is needed in the field of amblyopia. Patients can seek help in a timely manner only if there is public awareness. Often, the patient is young and not able to articulate his visual complaints. The parents may also be young and not aware of vision research and brain plasticity.

ChatGPT-3.5 (OpenAI) is a free and advanced large language model (LLM) launched on November 30, 2022. By December 4, 2022, it had over 1 million users, and by the 2-month mark, the monthly active users had been reported to exceed 100 million [6]. The demographics of ChatGPT users are those of the internet user population. The average age ranges between 18 and 29 years, matching the ages of young parents with amblyopic children. [7]. ChatGPT-3.5 has natural language processing capabilities and uses artificial intelligence (AI) to train in the auto-completion of sentences and ideas.

Health literacy may be a problem for ChatGPT-3.5 and patient users [8-10]. ChatGPT-3.5 could aid in patient education, but it types out texts in advanced high-school to college-level English. An estimated 130 million Americans lack proficiency in literacy, essentially reading below the equivalent of a sixth-grade level [9]. The average American reads between seventh and eighth grade [10,11]. Health literacy is a recognized problem for most Americans [11-13].

Can ChatGPT and Google Assistant be a source of patient education for our patients with amblyopia? Is it accurate and is it understandable [14,15]? Our study investigates whether an LLM and a voice assistant can adhere to the American Association for Pediatric Ophthalmology and Strabismus (AAPOS) guidelines for patient education. The AAPOS is an authoritative organization that provides educational information to the public and ophthalmologists about various eye diseases in children [16]. There is patient information for many eye diseases including amblyopia on the AAPOS website [16]. No institutional review board approval was required because no patient participants or patient data were involved in this study.

## Methods

In AAPOS’ “Learn More About Eye Conditions” section, there is a glossary of terms for “Amblyopia” [16]. From that hyperlink, we found the AAPOS’ 4 questions to query ChatGPT-3.5 and Google Assistant and then subsequently recorded their responses. The embedded video was transcribed using the “closed caption” option. From this point onward, our usage of the term AAPOS will refer to the section of the AAPOS website containing information on amblyopia. From AAPOS, 2 ophthalmologists (GW and DAL) chose the keywords within the text. The text was transcribed in Google Docs and included in the keywords.

We chose the ChatGPT-3.5 version because it is the free and most widely used version. The more advanced ChatGPT-4.0, released on March 13, 2023, requires a paid subscription. The four questions from the AAPOS website on “amblyopia” are as follows: (1) What is amblyopia? (2) What causes amblyopia? (3) How is amblyopia treated? (4) What happens if amblyopia is not treated? [16]. To assess the chatbots’ responses, we used the Flesch-Kincaid Grade Level test. This test has been used by the US Navy since the 1970s [17]. In addition, Flesch-Kincaid has also been used for scoring the results as shown by Lee et al [18] and the Pennsylvania Department of Insurance [19]. It has also been used in the Pennsylvania Department of Insurance to ensure readability [20].

For scoring the reading level, we used the “Flesch–Kincaid Grade Level Formula,” which presents a score as a US grade level [17]. This test is used in the US Navy and the US Department of Education to allow teachers, parents, librarians, and others to judge the readability level of schoolbooks and textbooks [17]. The formula yields the “mean number of years of education” generally required to understand the book, text, or assigned reading. The resultant score (grade level) is particularly relevant when the number is greater than the tenth grade. The formula for Flesch-Kincaid is as follows [21]: Grade Level=0.39*(words/sentences)+11.8*(syllables/words)15.59. The responses of AAPOS, ChatGPT-3.5, and Google Assistant to the questions were pasted into the Flesch-Kincaid Readability Calculator to obtain the readability grade levels [22]. For the word count measurement, we used Google Docs tools. Keywords from the AAPOS website were used as a reference point to score the ChatGPT-3.5 and Google Assistant responses. A total of 26 terms were used in the 4 questions from the AAPOS website.

## Results

For the 4 responses, ChatGPT used “eye doctor” once and never used “ophthalmologist”. Meanwhile, Google Assistant used “ophthalmologist” once, and AAPOS used the term twice, while neither used “eye doctor” (Table 1). Giving weighted scores to the individual keywords for the 4 questions, ChatGPT scored 47% in keyword usage, while Google scored 46%.

**Table 1:** Comparison of keywords.

Comparison of keywords													Weighted score						
Keywords		CG^a^	GA^b^	AAPOS^c^	CG total, n/N		GA total, n/N		AAPOS total, n/N		Points			CG points, n/N		GA points, n/N		AAPOS points, n/N	
**Question 1**						3/7		2/7		7/7		N/A^d^			12/25		8/25		25/25
	Lazy eye	✓	✓	✓							4								
	Brain	✓	✓	✓							4								
	High refractive error			✓							3								
	Strong glasses			✓							3								
	Early childhood or young age			✓							3								
	Vision loss	✓		✓							4								
	Abnormal vision development			✓							4								
**Question 2**						4/11		0/11		11/11		N/A			8/25		0/25		25/25
	Eye crosses			✓							3								
	Eye drifts out			✓							3								
	Brain avoids double vision			✓							2								
	Suppressing the vision			✓							3								
	Cataract	✓		✓							2								
	Drooping eyelid			✓							2								
	No clues			✓							2								
	Pediatrician			✓							2								
	Deprivation	✓		✓							2								
	Strabismus	✓		✓							2								
	Refractive	✓		✓							2								
**Question 3**						3/4		4/4		4/4		N/A			19/25		25/25		25/25
	Glasses or contact lenses	✓	✓	✓							7								
	Patches or patching	✓	✓	✓							7								
	Surgery		✓	✓							6								
	Drops	✓	✓	✓							5								
**Question 4**						1/4		2/4		4/4		N/A			8/25		13/25		25/25
	9-10 years (cut off for effective treatment)			✓							7								
	Pediatric ophthalmologist			✓							5								
	Permanent vision decrease or loss	✓	✓	✓							8								
	Ophthalmologist		✓	✓							5								
Total keyword appearance						11/26		8/26		26/26		Percentage of points earned			47/100		46/100		100/100

^a^CG: ChatGPT.

^b^GA: Google Assistant.

^c^AAPOS: American Association for Pediatric Ophthalmology and Strabismus.

^d^N/A: not applicable.

As seen in Table 2, AAPOS displayed the lowest reading comprehension level necessary among the 3 sources (mean 11.4, SD 2.1), whereas Google Assistant showed the highest level (mean 13.1, SD 4.8). ChatGPT displayed an average reading grade level of 12.4 (SD 1.1). In performing statistical *t* tests between the Flesch-Kincaid grades of the questions, we see that none of the responses varied in reading level significantly from one another. See footnotes under Table 2, where the *P* values are not significant. However, the word counts of the responses, as seen in Table 3, were significantly different between AAPOS and Google Assistant, as well as between ChatGPT and Google Assistant (*P*=.57); AAPOS versus Google Assistant (*P*.001); and ChatGPT versus Google Assistant (*P*.001). See footnotes under Table 3, where the *P* values are significant.

For question 4, ChatGPT’s answer is misleading. It cites age 6 years as the preferable age to start treating amblyopia. Effective intervention in amblyopia can start as early as possible and even as late as age 9 or 10 years, according to AAPOS. Recovery of vision at any age is considered by ophthalmologists to be beneficial in terms of quality of life. The subtleties are missing in the ChatGPT answers (Tables S1-S3 in Multimedia Appendix 1). In addition, there are errors in omission when not mentioning the need to see an ophthalmologist, pediatric ophthalmologist, or pediatrician.

**Table 2:** Flesch-Kincaid Grade Level^a^.

Information sources	Flesch-Kincaid Grade Level				
	Question 1	Question 2	Question 3	Question 4	Mean (SD)
ChatGPT	11	12.6	12.5	13.6	12.4 (1.1)
AAPOS^b^	9.5	10.4	14.3	11.4	11.4 (2.1)
Google Assistant	10.7	7	15.7	13.3	13.1 (4.8)

^a^*P* values of *t* test: AAPOS versus ChatGPT: *P*=.42; AAPOS versus Google Assistant: *P*=.90; ChatGPT versus Google Assistant: *P*=.71.

^b^AAPOS: American Association for Pediatric Ophthalmology and Strabismus.

**Table 3:** Word count^a^.

Information sources	Word count				
	Question 1	Question 2	Question 3	Question 4	Mean (SD)
ChatGPT	167	188	228	164	186.8 (29.5)
AAPOS^b^	110	172	77	63	105.5 (48.5)
Google Assistant	44	56	38	46	46 (7.5)

^a^*P* values of *t* test: AAPOS versus ChatGPT: *P*=.57; AAPOS versus Google Assistant: *P*.001; ChatGPT versus Google Assistant: *P*.001.

^b^AAPOS: American Association for Pediatric Ophthalmology and Strabismus.

## Discussion

During the past 2 years of the COVID-19 pandemic, many patients self-isolated or were afraid to go to their doctor’s appointments for eye care. Many of their family members used the internet or voice assistants for patient information. The natural language model in ChatGPT met an unspoken need [23-26]. By 2023, there were 100 million ChatGPT users from all over the world who accessed this natural language model. Google Assistant was first launched in May 2016, and has the largest share of the voice assistant market. Google Assistant uses Google’s AI but relies on search engine optimization since it lists snippets of phrases from websites in its citations [27]. While the answers are short for Google Assistant (they are succinct and to the point), they are a shorthand complement to ChatGPT’s long-winded answers.

ChatGPT may play a role in patient education but when compared with AAPOS, the LLM mentions “eye doctor” once and not “ophthalmologist”, whereas Google Assistant does mention “ophthalmologist.” Both ChatGPT and Google Assistant mention “lazy eye” and “brain” which is excellent. The 3.5 version of ChatGPT was trained on the internet data through the year 2021, but in the future, more advanced versions of ChatGPT may use data post 2021.

We are unclear about the training data sets for these LLMs. There are 6 million people with ophthalmic diseases in America, while those with hypertension are a total of 90 million; thus, ChatGPT’s ability to recognize ophthalmic terminology may be more limited. More patients are asking questions about common American afflictions such as heart disease, stroke, and diabetes than eye diseases.

One of the disadvantages of AAPOS, Google Assistant, and ChatGPT’s text responses is that all of these modalities require a high reading level, greater than the seventh- to eighth-grade reading level of the average American [9]. Patients with amblyopia who are more educated will be able to understand that partial loss of vision is intertwined with brain development. The ability to process these ideas may require higher health literacy and higher education levels than most young parents.

Our patients from diverse backgrounds in the United States may not be able to understand ChatGPT, AAPOS, and Google Assistant. The “Public & Patients” section on the American Academy of Ophthalmology website has material written at the seventh- or eighth-grade level and the answers are less detailed than those on the AAPOS website [16].

Another potential problem that might arise with the use of LLMs is that the natural language is set in a conversational tone, thus minor flaws such as “go see a doctor” may seem perfectly normal. ChatGPT and Google Assistant have information about amblyopia for the lay public. None of the answers tell patients about the urgency or need to see a pediatric ophthalmologist. This is due to the responses created by the prompt engineers who manage the chatbots’ answers. In 2024, all ChatGPT responses have attached statements such as, “please see a medical professional.”

In the spring of 2023, there were new competitors to ChatGPT. Bard (currently known as “Gemini”) and PaLM2 are both Google-directed AI LLMs [27-29]. Bard was launched briefly in March 2023 and May 2023, with mixed results in Europe. However, in mid-August 2023, it was launched in the United States. Its algorithms are augmented with those created under PaLM2 [28,29]. As of this writing, Bard was replaced by Gemini on February 8, 2024.

PaLM2, which powers Gemini, has 160 languages and the future capability of “deductive reasoning” [28,29]. Gemini is part of the powerful Google search engine, which can access the user’s past history of queries. Thus, Gemini can provide a tailored response for the user. Whether this will make Gemini as popular as ChatGPT, or not, is unknown since it is the latest LLM for Google.

OpenAI has ChatGPT and Microsoft has Copilot. Microsoft owns a large stake in OpenAI, the company that first launched ChatGPT. All the chatbots are free and easy to use, which will make them a source of health information.

Our patients have access to mobile apps and the internet, ChatGPT, Google Assistant, Gemini, and more AI chatbots. They can compare different answers which may be the solution for free patient education.

The use of these free chatbots can save the pediatric eye clinic time and money as resources become scarce with increasing patient load and electronic chart documentation demands. A medical assistant is paid US $38,000 per year in the continental United States [30]. An orthoptist has an average annual salary of US $90,000 [30]. The cost of ChatGPT 3.5 is zero. One can imagine the cost savings of 1 less medical assistant with full-time benefits versus a free Chatbot. At most, the paid version of ChatGPT 4.0 is US $20 per month. The cost savings are an incentive for all pediatric eye and general ophthalmology offices to be aware of this free resource. The ease of use will allow Open AI, the creator of ChatGPT, to gain market share and crush its competitors.

As physicians, we can assign medical assistants to show ChatGPT to selected patients with “lazy eyes.” In some cases, patients want reassurance and repeated explanations of “what is amblyopia.” In such cases, these chatbots and voice assistants are a cost-effective means of public education. They are useful to our patients with poor access to physicians.

In conclusion, the age of AI-mediated technology for patient education has arrived. The latest LLM is available and provides cheap and free patient education in office settings [24,29,30]. The LLMs may communicate possible minor inaccuracies and biases to the LLMs and Google Assistant will improve their “training.” ChatGPT is already partnering with the Mayo Clinic and Stanford for conversational response emails to patients. There is ambient natural language processing now that will allow us to “talk” to our patients and our notes will be immediately transcribed into our electronic health records.

In 2024, the LLM chatbots will provide users with clear warning messages when asking about medical conditions. Other chatbots will provide web resources from clinicians that meet current medical guidelines. This is especially important since these models do not have access to the patient’s medical history and cannot fully understand the complexity of a patient’s health situation [31].

As ophthalmologists, we have the power to change ChatGPT and future LLMs through their “training” on our websites and journals. In fact, in the user’s account, repeated questioning will “train” ChatGPT in 2024. This is new as of 2024. Individually as physicians, we can retype “see an ophthalmologist or pediatric ophthalmologist” as a necessary sentence fragment for our ophthalmic websites and web journals. From May 2023, to now, the current questions and answers have evolved in ChatGPT and the other chatbots to include phrasing that they are not physicians. There is evidence that the training is changing and by the time this paper is published, the AI-mediated chatbot answers will have improved with its million queries per day.

Patients and physicians may need Google Assistant, ChatGPT, and similar AI chatbots. We hope to harness the abundance of accessible information provided by LLMs to guide our patients’ journey toward improved vision.

## Appendix

Multimedia Appendix 1 ChatGPT responses.

## Abbreviations

AAPOS: American Association for Pediatric Ophthalmology and Strabismus
AI: artificial intelligence
LLM: large language model
