# Supplementary material for: ChatGPT and Google Assistant as a Source of Patient Education for Patients With Amblyopia: Content Analysis
Source: J Med Internet Res. 2024 Aug 15;26:e52401. doi: 10.2196/52401 (PMC11362700; doi:10.2196/52401)
Supplement: Multimedia Appendix 1 [file jmir_v26i1e52401_app1.docx]

**Table S1**. ChatGPT Responses to Questions.

| 1. What is amblyopia? |
| --- |
| Amblyopia, also known as lazy eye, is a condition that occurs when one eye does not develop normal vision during childhood. This happens because the brain receives different visual signals from the two eyes and learns to rely on the stronger eye. As a result, the weaker eye is not used properly and its vision does not develop properly.  Amblyopia is a common condition, affecting about 2-3% of children. It usually develops before the age of 6 and can be caused by a variety of factors, including strabismus (misalignment of the eyes), unequal refractive errors (one eye has a different prescription than the other), or visual deprivation (e.g. cataract).  If left untreated, amblyopia can cause permanent vision loss in the affected eye. However, early detection and treatment can often restore normal vision. Treatment usually involves correcting any underlying refractive errors, using an eye patch to encourage use of the weaker eye, or in some cases, using eye drops to blur the vision in the stronger eye. |
| 2. What causes Amblyopia? |
| Amblyopia, also known as lazy eye, can be caused by several factors that affect the development of normal vision in childhood. The most common causes of amblyopia are:   1. Strabismus: When the eyes are not properly aligned and point in different directions, it can cause the brain to receive different visual information from each eye. The brain then learns to ignore or suppress the information from one eye, leading to amblyopia. 2. Refractive errors: When one eye has a different prescription than the other eye, such as nearsightedness, farsightedness, or astigmatism, it can cause the brain to receive different visual information from each eye. If not corrected, this can lead to amblyopia in the weaker eye. 3. Visual deprivation: When something blocks or obstructs the vision in one eye, such as a cataract or ptosis (droopy eyelid), it can cause the brain to receive less visual information from that eye, leading to amblyopia.   It is important to detect and treat amblyopia as early as possible, ideally before the age of 6, when the visual system is still developing. If left untreated, amblyopia can cause permanent vision loss in the affected eye. |
| 3. How is amblyopia treated? |
| The treatment for amblyopia, or lazy eye, depends on the underlying cause of the condition. In general, the goal of treatment is to strengthen the weaker eye and encourage the brain to use both eyes together. The earlier the treatment is started, the better the chance of restoring normal vision.  The most common treatments for amblyopia are:   1. Corrective lenses: If the amblyopia is caused by a refractive error, such as nearsightedness, farsightedness, or astigmatism, correcting the vision with glasses or contact lenses may be all that is needed to treat the condition. 2. Patching therapy: Covering the stronger eye with an eye patch for several hours a day can help encourage the weaker eye to work harder and develop better vision. The length and frequency of patching depends on the severity of the amblyopia and is determined by an eye doctor. 3. Atropine eye drops: In some cases, using atropine eye drops in the stronger eye can temporarily blur the vision and encourage the weaker eye to work harder. 4. Vision therapy: This involves a series of exercises and activities designed to improve visual acuity, depth perception, and eye coordination.   In more severe cases of amblyopia, a combination of these treatments may be needed. It is important to have regular follow-up visits with an eye doctor to monitor the progress of treatment and ensure that the condition is being properly managed. |
| 4. What happens if amblyopia is not treated? |
| If amblyopia, or lazy eye, is left untreated, the weaker eye can continue to develop poor vision, leading to permanent vision loss. This occurs because the brain learns to rely on the stronger eye and suppresses or ignores the visual information from the weaker eye. Over time, the connections between the brain and the weaker eye can become weaker, making it more difficult to restore normal vision.  Amblyopia can also affect depth perception, eye-hand coordination, and visual processing skills. Children with amblyopia may have difficulty with tasks that require good vision in both eyes, such as reading, sports, or driving. In addition, amblyopia can affect self-esteem and social interactions, especially if the condition is noticeable or affects appearance.  It is important to detect and treat amblyopia as early as possible, ideally before the age of 6, when the visual system is still developing. If amblyopia is detected and treated early, there is a good chance of restoring normal vision and preventing permanent vision loss. |

**Table S2**. Google Assistant Responses to Questions.

| 1. What is amblyopia? |
| --- |
| Amblyopia, also called lazy eye, is a disorder of sight in which the brain fails to fully process input from one eye and over time favors the other eye. It results in decreased vision in an eye that typically appears normal in other aspects. |
| 2. What causes amblyopia? |
| It develops when there’s a breakdown in how the brain and the eye work together, and the brain can’t recognize the sight from 1 eye. Over time, the brain relies more and more on the other, stronger eye — while vision in the weaker eye gets worse. It’s called “lazy eye” because the stronger eye works better. |
| 3. How is amblyopia treated? |
| Treatment consists of devices: Treatment includes eye patches, drops, glasses or contact lenses, and sometimes surgery.  Devices: Bifocals, Corrective lens, Contact lenses, and Occlusion therapy  Medications: Involuntary nervous system blocker  Surgery: Eye surgery  Specialists: Optometrist, Pediatrician, and Ophthalmologist. |
| 4. What happens if Amblyopia is not treated? |
| If amblyopia is not treated early, specifically during the period of visual development between birth and 7 years of age, it can result in a permanent visual defect or loss of depth perception. If you suspect lazy eye in your child, consult a doctor right away. |

**Table S3**. AAPOS Responses to Questions

| 1. What is amblyopia? |
| --- |
| [In this webcast we hope to provide you with a better understanding of what amblyopia is and how we treat it. amblyopia is often referred to as lazy eye by many people but a better term would be lazy vision. amblyopia is a medical term that either describes the loss of vision in  because it is being ignored by the visual part of the brain or a decrease in vision in both eyes because of a very high refractive error which causes someone to need a strong glasses prescription. Amblyopia always begins in early childhood and is best treated effectively at a young age.]  Amblyopia is decreased vision in one or both eyes due to abnormal vision development in infancy or childhood. In the first few years of life, the brain must learn to see or interpret the images provided by the eyes. In amblyopia, the brain receives a poor image from the eye and thus does not “learn to see well. Vision loss occurs in this case because nerve pathways between the brain and the eye are not properly stimulated.  In amblyopia, there may not be an obvious problem of the eye. Another word for amblyopia is often “lazy eye.” It is the leading cause of vision loss amongst children. |
| 2. What causes amblyopia? |
| [There are many causes of amblyopia sometimes there is an eye muscle control problem and one eye crosses in or drifts out in young children the brain avoids double vision by ignoring or suppressing the vision of one eye there can be structural problems with an eye including a cataract or drooping eyelid that actually blocks the vision from entering the eye properly. Oftentimes there are no clues that a child has amblyopia.]  Normal vision develops during the first few years of life. At birth, infants have very poor vision. As infants grow and use their eyes, the vision improves as the vision centers in the brain develop. If children are not able to use their eyes, the vision centers in the brain do not develop properly and the vision is decreased. Amblyopia may occur despite normal appearance of the eye structures.  The most common cause is refractive error in one or both eyes that is not corrected early in childhood resulting in poor development of the visual function in the affected eye(s). This is called refractive amblyopia. Another common cause is strabismus or eye misalignment. This is called strabismic amblyopia. Rarely there is a structural anomaly that impairs vision like a droopy eyelid or an opacity in the eye, such as a cataract or corneal scar. This is called deprivation amblyopia.  The three types of amblyopia, strabismic amblyopia, refractive amblyopia, and deprivation amblyopia, may occur at the same time in a single eye. |
| 3. How is amblyopia treated? |
| [A common cause of amblyopia occurs when two eyes have different focus powers they cannot work together and one eye is ignored unless a child wears glasses full time. Often children do not realize they cannot see as well out of one eye until a vision screening test at school or the pediatrician's office detects a problem when both eyes have amblyopia it is because both eyes have a very strong need for glasses the treatment for amblyopia first depends on the underlying cause treatment often requires glasses and some problems might require surgery.]  The treatment depends on the type of amblyopia present. Refractive and some forms of strabismic amblyopia are treated with glasses and/or contact lenses. Some forms of strabismic and deprivation amblyopia are treated with surgery (for example, by removing a cataract). If the amblyopia is only in one eye or much worse in one eye, amblyopia is treated by encouraging the child to use the weaker eye via patching or eye drops that blur the better-seeing eye. |
| 4. What happens if Amblyopia is not treated? |
| [Most importantly treatment of the weaker eye requires patching or eye drops to blur the vision in the stronger eye this allows the vision and the weaker eye to catch up usually vision is most effectively improved up until the age of 9 to 10. If treatment is done as recommended, improvement after this age is still possible but it is less likely to normalize the vision because the brain doesn't respond as well to treatment. This is why it is so important to discover and treat amblyopia at a young age. For more information, you can look at www.aapos.org or ask your pediatric ophthalmologist]  If amblyopia is not treated in childhood, the vision in the affected eye(s) will be permanently decreased. Our current treatments for amblyopia are not effective in adults.  For most children, the ophthalmologist will give the instructions and monitor the progress, but the patient and the family will do the hard work of actually performing amblyopia treatment by patching, glasses or eye drops. |

*Brackets [ ] indicate the transcription of the captions from the informational video provided on the AAPOS.org amblyopia page.
